# 2D-shear wave elastography in the evaluation of suspicious superficial inguinal lymph nodes: Reproducibility and region of interest selection

**DOI:** 10.1371/journal.pone.0265802

**Published:** 2022-03-28

**Authors:** Olli Lahtinen, Mika Pulkkinen, Reijo Sironen, Ritva Vanninen, Suvi Rautiainen

**Affiliations:** 1 Diagnostic Imaging Centre, Department of Clinical Radiology, Kuopio University Hospital, Kuopio, Finland; 2 Institute of Clinical Medicine, Unit of Radiology, University of Eastern Finland, Kuopio, Finland; 3 Institute of Clinical Medicine, Clinical Pathology, University of Eastern Finland, Kuopio, Finland; 4 Department of Clinical Pathology, Kuopio University Hospital, Kuopio, Finland; 5 Institute of Clinical Medicine/Clinical Pathology, University of Eastern Finland, Kuopio, Finland; 6 Cancer Centre of Eastern Finland, Kuopio, Finland; Universita Politecnica delle Marche, ITALY

## Abstract

**Purpose:**

To assess the ability of 2D-Shear wave elastography (2D-SWE) to evaluate its reproducibility, to define the optimal orientation and size of the region of interest (ROI), and to differentiate benign from malignant inguinal lymph nodes (LNs).

**Method:**

Thirty-two suspicious inguinal LNs from 21 patients were evaluated with 2D-SWE. SWE measurements were obtained in two orthogonal planes. To investigate reproducibility, sensitivity and specificity, circular ROIs with a diameter of 1 mm, 2 mm, 3 mm and 5 mm were placed on the cortex of the LNs. Additionally, one freehand ROI was drawn covering majority of the LN. Two observers performed five sets of SWE measurements for each ROI size. All LNs underwent core needle biopsy or were surgically removed.

**Results:**

The 3 mm ROI for Mean-E in axial plane showed high interrater agreement [intraclass correlation coefficient (ICC) 0.899] with the cut-off value of 7.31 kPa resulting in 88.9% sensitivity and 60.9% specificity for differentiating malignant from benign LNs. In benign LNs, mean elasticity of the ROI was lower (7.68 ± 3.82 kPa; range, 3.41–15.40 kPa) compared to the malignant LNs (15.81 ± 10.61 kPa; range, 3.86–36.45 kPa).

**Conclusions:**

The most reproducible way to measure stiffness in inguinal LNs is a 3 mm circular ROI centered on the cortex of the LN in axial plane. Elasticity values were higher in the malignant LNs reflecting the stiffer nature of the metastatic LNs. 2D-SWE offers a noninvasive ultrasonographic tool to assess superficial inguinal lymph nodes with high reproducibility.

## Introduction

When a malignancy is detected, accurate assessment of the regional lymph nodes (LNs) is crucial for the management and prognosis of the patient. Superficial LNs are easily detected and evaluated with ultrasound (US). US B-mode alone has sensitivity of 61.4–98.0% and specificity of 31.4–97.0% in the differentiation of malignant and benign LNs in superficial lymph node regions [1–6]. Despite its high resolution in superficial areas none of the US criterions can be used as a sole predictor of malignancy and LN biopsy remains to be the golden standard for the characterization of the LNs [7–11].

US elastography has been available since 1990s [12, 13]. Currently, there are two kinds of elastography methods: strain and shear wave elastography (SWE). SWE using mechanical shear waves improves the reproducibility of the tissue elasticity measurements, since the measurement of the velocity of the shear waves gives quantitative and qualitative results from the elasticity of the tissues [7, 12, 13]. Newest application of shear wave imaging is the 2D-SWE. Unlike the older shear wave imaging methods, the 2D-SWE uses acoustic radiation force in multiple points allowing real time monitoring of shear wave propagation. Promising results to differentiate malignant from benign superficial LNs have been reported using SWE [7, 9, 14, 15].

Like conventional B-mode US, elastography has its limitations. Anisotrophy, depth of the measurement, tissue interfaces, attenuation of the signal, tissue pulsations due to blood pulsation, breathing and operator dependent high contact force may affect the reliability of SWE [13, 16–19].

Different sizes and shapes of regions of interest (ROIs) have been investigated mostly in elastography of breast lesions. Yet, there is no consensus on the optimal type, size or plane of the ROI for the SWE measurements. Typically, a small 2–3 mm circular ROI is placed over the stiffest part of the lesion on the axial plane. In contrast, also larger ROIs covering the whole lesion and two orthogonal planes have been used [20–27]. Thus far, no recommendations exist for ROI types in LN assessment with SWE.

The aims of the present validation study were to evaluate the reproducibility of 2D-SWE and to define the optimal ROI size for inguinal LNs. As a secondary endpoint, we wanted to study whether the depth, anisotrophy of the tissue or vicinity of the femoral artery would affect the repeatability of the measurements. Additionally, we wanted to assess the ability of 2D-SWE to differentiate benign and malignant inguinal LNs.

## Materials and methods

### Patients and study design

Approval of institutional review board, local ethics committee and written informed consent of the patients were obtained for this prospective single center study.

All consecutive patients who were scheduled for inguinal US as part of their preoperative staging examinations or had otherwise a suspicion of abnormal LNs in the inguinal region were invited to participate in this study between November 2016 and September 2018. Exclusion criteria were as follows: 1) patient could not provide informed consent 2) inguinal LNs less than 5 mm on their short axis to avoid surrounding tissues to be included in larger SWE ROIs 3) inguinal LNs without further histopathological confirmation. Biopsies were performed, when necessary depending on the clinical setting and were taken after the SWE examinations to avoid effects of possible hemorrhage on the measurements.

### Inguinal US and elastography

The US examinations were performed independently by one of two radiologists with 5 and 15 years of experience in conventional US and 3 years of experience in SWE. All studies were performed with Logiq E9™ US-device (GE Healthcare, Chicago, USA) with ML6-15 (4–15 MHz) linear array transducer for grey scale imaging. SWE was performed with 2D wide band linear transducer, 9L (2–8 MHz).

During the US studies, the patients rested in supine position on the examination table. Inguinal region and the LNs were first examined with traditional grey scale US. The size, short axis, cortical thickness, shape and vascular pattern of the LNs were registered. A LN was considered abnormal if it showed rounded shape, no hilar fat, cortical thickness greater than 3 mm or lobular cortical thickening, mixed or cortical vascularity, or the short axis > 1 cm. If a LN greater than 5 mm in short axis was found in the grey scale US, 2D-SWE was performed. Furthermore, the distance between the SWE measured LN and femoral artery and skin were measured.

Based on the grey scale US, SWE was conducted on each inguinal side to the largest LN or to the LN with most abnormal features if a suitable LN was found. A thick layer of gel was put on the skin and the probe was placed on a perpendicular plane to the skin. A minimal external force was applied to the probe avoiding too high compression against the tissue. SWE measurements were acquired and registered in two perpendicular planes to the LN. First a longitudinal view along the LN’s long axis was acquired (called axial plane in further text), followed by the probe rotation of 90 degrees to have a transverse image (called sagittal plane in further text).

**Fig 1:**
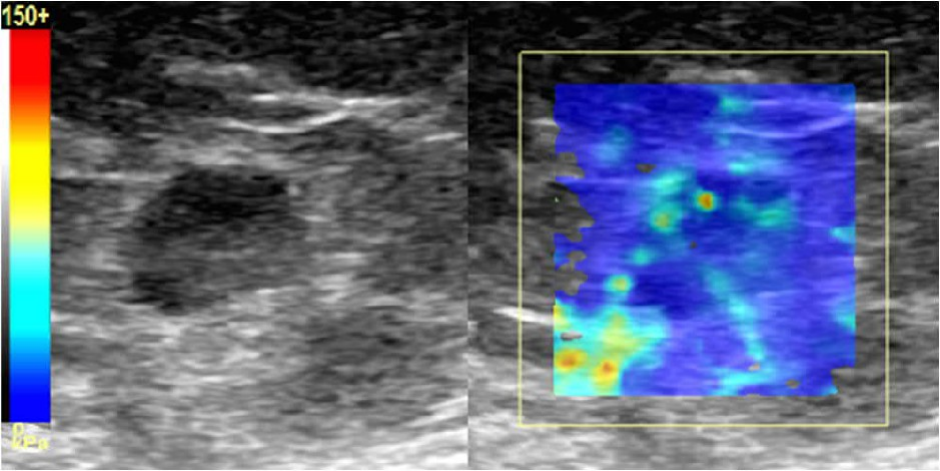
B-mode and elastography images in axial plane of an inguinal lymph node (LN) from a 68-year old patient with vulvar cancer. Although the B-mode image shows a slightly rounded LN, elastography shows the LN as blue (soft) indicating benign etiology. In histopathology, the inguinal lymph nodes proved to be benign.

**Fig 2:**
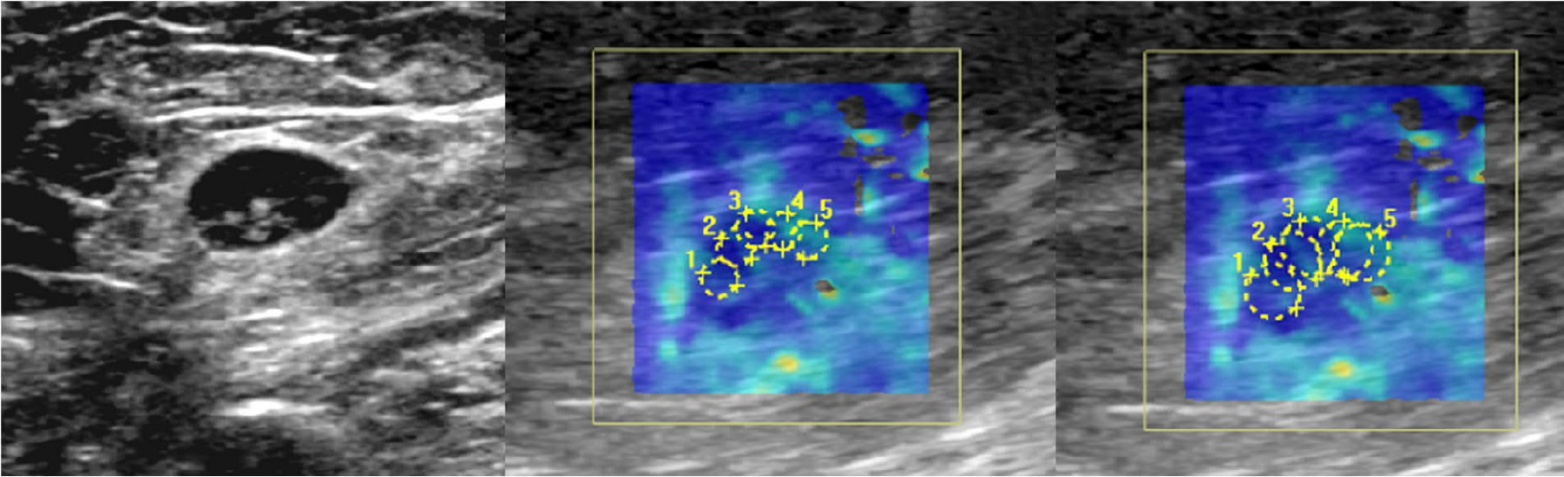
SWE measurements with different ROI sizes in axial plane. A 7 mm inguinal LN of a 70-year old woman with vulvar cancer is shown in B-mode image (left). The same LN is illustrated with five 2 mm (middle image) and 3 mm (right image) SWE ROIs. ROIs are centered evenly on the 4 mm cortex to cover majority of it. The Mean-E values were 7.8 kPa and 7.0 kPa with 2 mm and 3 mm ROIs accordingly. The LN proved to be histopathologically benign.

The more detailed SWE measurements with several ROI types were later performed blindly and independently by two radiologists with 5-year experience in US and 3 years of experience in SWE. SWE sampling box was fixed to 15 x 15 mm. Color-coded elastography maps showed stiff areas (high kPa) as red and soft areas (low kPa) as blue. The color-coded SWE image was superimposed onto the conventional grey scale B-mode image and displayed in a split screen together with normal B-mode image (Fig 1). Circular ROI was centered to cover the cortex of the LN or the cortex and the hilum if the ROI did not fit purely in the cortex. ROI diameters of 1, 2, 3 and 5 mm were used in the analysis (Fig 2). The US device provided a fixed ROI size of 5 mm, smaller ROIs were calculated individually from the surface area given by the device to match the smaller ROI sizes. A total of five ROIs with diameters of each predefined size were placed with even spacing to cover the cortex in both perpendicular planes. In addition, a manual freehand circular ROI, covering most of the LN, was also drawn once. SWE data was deemed sufficient when majority of the color box was color-coded (>75%) and the cycles remained constant to avoid excess use of pressure on the probe. Approximately five images were taken with each orientation. The elasticity (E) of the ROI was calculated by the system representing the average elasticity value of the pixels in the ROI. Mean elasticity (Mean-E) was defined as the mean value of the five measurements. Additionally maximum (Max-E) and minimum (Min-E) elasticity out of the five ROI measurements were registered. Due to software update of the US device during the study all of the elasticity indices were not automatically included in the measurements after the update. Thus, to keep the data homogeneous some of the elasticity indices could not be used.

### Patient treatments

All patients received standard treatment according to national and local guidelines regardless of the findings in SWE. All patients in this study had histological verification through a core needle biopsy (CNB) or surgical removal of LN(s) as a part of their normal treatment. CNB was performed using a 16 or 14 gauge needle (Temno Evolution™). A total of 3–7 biopsies were obtained depending on the clinical setting. At least six samples were taken if lymphoma was suspected. Clinical 6-month follow up period was included in the study setting.

### Histopathology

**Fig 3:**
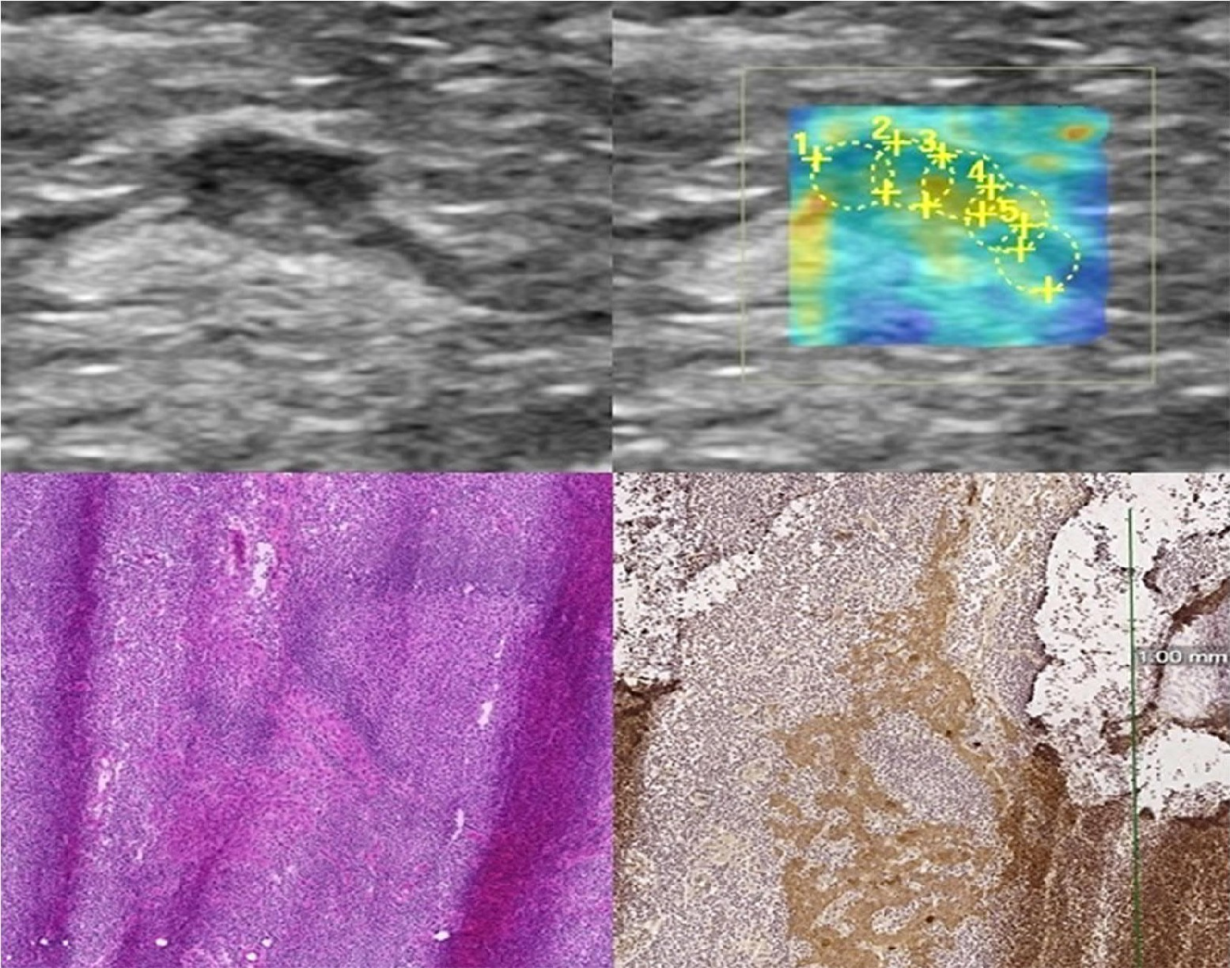
Ultrasound images and histopathology of a micrometastatic inguinal LN of a 67-year old woman with vulvar cancer. B-mode and elastography images (upper images) illustrate a superficial inguinal LN with abnormal focal cortical thickening. SWE demonstrated Mean-E value of 9.68 kPa and Max-E value of 11.46 kPa with 3 mm ROI in the axial plane. Histopathological analysis revealed a micrometastatic 1 mm deposit of squamocellular carcinoma. HE and cytokeratin stainings are illustrated in the lower row. atypical nuclei show variation in their size and shape. Additionally metastatic cell nuclei stain positive (brown) in cytokeratin staining (scale bar 1 mm).

CNB samples and surgical specimens were examined histopathologically in pathology laboratory as a part of the normal diagnostic procedure. The samples were sectioned into 4 μm thick slices after formalin fixation and paraffin embedding. Standard hematoxylin and eosin (HE) staining was done to all LNs. Additionally, immunohistochemical stainings (cytokeratins) were performed whenever found necessary (Fig 3).

### Statistical analysis

Statistical analysis was performed using statistical software (SPSS Version 22.0, SPSS, Chicago, Illinois). Continuous variables with normal distribution are reported as mean ± standard deviation. A power calculation was used to verify statistically significant sample size for this reproducibility study (α = 0.05, power 0.80). The ROI size to best differentiate benign LNs from the malignant LNs was selected by using the areas under the curve (AUC) values from the receiver operating characteristic (ROC) curve analysis. Delong test was used to compare the ROC curves. McNemar test was applied to compare the diagnostic performance of the different ROI types.

Interrater agreement between the two readers was assessed by intraclass correlation coefficient (ICC), using the mean value of the five measurements of each observer. The ICC values were considered excellent (> 0.90), good (0.75–0.90), moderate (0.50–0.75) or poor (< 0.50). *P* values for the ICCs of the different ROIs were calculated by using the bootstrap method. Paired samples t-test was used to calculate statistical significances for the ICC values, *P* < 0.05 was considered statistically significant.

## Results

Altogether 27 consecutive patients with 39 inguinal LNs were initially recruited to this study. Data from 5 LNs was lost due to US device software updates. Additionally, one LN with measurements in only one plane and one LN with no histopathological verification were excluded. After exclusion criteria, altogether 32 inguinal lymph nodes from 21 patients were included in the statistical analysis. Mean age of the patients was 68 years (range 27–85 years). Majority of the patients had a malignant disease treated (19/21, 90.5%) of which vulvar cancer was the most common (16/20, 80.0%). No malignancy was detected after 6-month follow up in patients who had no malignancy in the final histopathology of the inguinal LN. Patient demographics and LN characteristics from grey scale US are presented in Table 1.

**Table 1:** Patient demographics and lymph node (LN) characteristics.

Patients and characteristics	N (%)	Mean (range)
N	21	
Gender	Male 4 (19.0) Female 17 (81.0)	
Age (years)		68 (27–85)
BMI		33.9 (19.1–44.6)
**Number of LNs evaluated/patient**		
1	10 (47.6)	
2	11 (52.4)	
**LN**		**Median, (range) in mm**
Number	32	
Cortical thickness		4 (1–59)
Short axis		7 (5–59)
Depth		15 (3–27)
Distance from femoral artery		11 (2–21)

Majority (24/32, 75.0%) of the LNs were surgically removed while CNB was performed in 25.0% (8/32) of the LNs. Altogether, nine out of thirty-two (28.1%) of the LNs were malignant in final histopathology. Detailed histopathological findings are presented in Table 2. All malignant LNs expressed cortical thickness ≥ 5 mm is US.

**Table 2:** Histopathology of the core biopsy samples or removed inguinal lymph nodes (N = 32).

Histopathology	N (%)
Squamous cell carcinoma (SCC)	6 (18.7)
Serous ovarian cancer	1 (3.1)
Lymphoma	2 (6.3)
Reactive lymphoid tissue	2 (6.3)
Lymphoid tissue, no abnormal features	21 (65.6)

### Reproducibility

The reproducibility of the SWE measurements was highest in larger ROIs measured in axial plane; 5 mm ROI in Mean-E and 3 mm and 5 mm ROIs in Max-E (*P* = 0.003 and 0.006, respectively). In addition, the ICC scores were good to excellent (0.85–0.97) for Mean-E and Max-E measurements in axial plane (Table 3). In sagittal plane the ICC scores varied from poor to good (0.42–0.79).

**Table 3:** Intraclass correlation coefficient (ICC) for different regions of interest (ROIs).

ROI (size mm/type)	ROI plane	Mean-E (kPa) ± SD		Max-E (kPa) ± SD		Min-E (kPa) ± SD		ICC-values for Mean-E/Max-E/Min-E
		Observer 1	Observer 2	Observer 1	Observer 2	Observer 1	Observer 2	
1	Axial	9.32 ± 8.24	9.44 ± 7.08	18.63 ± 22.44	18.22 ± 17.46	4.49 ± 4.07	3.84 ± 2.66	0.87 / 0.85 / 0.61
2	Axial	9.80 ± 8.12	10.04 ± 9.93	17.10 ± 17.73	18.57 ± 23.88	5.01 ± 3.58	4.82 ± 4.15	0.91 / 0.90 / 0.70
3	Axial	9.96 ± 7.29	10.29 ± 9.50	16.84 ± 18.20	17.18 ± 20.59	5.82 ± 4.13	5.52 ± 5.04	0.90 / 0.96 / 0.82
5	Axial	9.95 ± 7.02	9.78 ± 7.80	14.20 ± 11.43	14.17 ± 14.06	6.69 ± 3.97	6.21 ± 4.47	0.97 / 0.95 / 0.91
1	Sagittal	12.32 ± 8.86	14.03 ± 10.74	21.15 ± 18.01	24.12 ± 19.40	6.08 ± 6.49	6.41 ± 5.43	0.70 / 0.42 / 0.65
2	Sagittal	12.29 ± 9.44	14.28 ± 10.98	21.57 ± 20.27	24.01 ± 19.32	6.24 ± 6.33	7.14 ± 6.77	0.77 / 0.72 / 0.91
3	Sagittal	12.28 ± 9.69	14.44 ± 11.31	19.76 ± 17.60	21.97 ± 19.15	7.01 ± 6.66	8.50 ± 6.29	0.74 / 0.74 / 0.77
5	Sagittal	12.19 ± 8.99	13.75 ± 10.25	16.58 ± 12.77	17.55 ± 13.24	8.58 ± 7.13	10.36 ± 8.15	0.79 / 0.79 / 0.80
Freehand ROI	Axial	9.61 ± 6.43	9.79 ± 6.60	-		-		0.95
Freehand ROI	Sagittal	11.46 ± 6.73	13.88 ± 10.50	-		-		0.61

In the reproducibility analysis the SWE measurements in axial plane yielded greater ICC-values than in sagittal plane (*P* = 0.006). Paired samples t-test showed no significant difference in the elastography analysis between the two observers. In addition, the distance from the LN to the femoral artery had no significant effect on reproducibility. However, depth of the LN had an influence on the ICC-values resulting in better reproducibility for more superficial LNs. Moreover, LNs with a cortical thickness greater than 3 mm had higher ICC values. Reproducibility in the subgroups is presented in Table 4.

**Table 4:** Effect of different parameters on reproducibility of SWE elastography, assessed by intraclass correlation coefficient (ICC) values.

Parameter	n	Mean-E ± SD Observer A	Mean-E ± SD Observer B	ICC-values	P-value
**All LNs**	32	10.92 ± 8.08	11.97 ± 9.47	0.80	0.971
**Plane**					0.006
**Axial**	32	9.73 ± 7.42	9.87 ± 8.18	0.87	
**Sagittal**	32	12.11 ± 8.74	14.07 ± 10.76	0.72	
**Distance from the femoral artery**					0.529
**< 10 mm**	16	12.98 ± 8.67	13.99 ± 9.54	0.77	
**≥ 10 mm**	16	8.86 ± 6.39	9.96 ± 8.59	0.81	
**Cortical thickness**					0.004
**≤ 3 mm**	14	8.85 ± 4.34	11.12 ± 8.08	0.57	
**> 3 mm**	18	12.53 ± 9.87	12.64 ± 10.40	0.93	
**Depth**					0.018
**< 15 mm**	14	14.23 ± 10.44	14.50 ± 11.63	0.92	
**≥ 15 mm**	18	8.35 ± 4.31	10.01 ± 6.61	0.61	
**Benign**	23	8.85 ± 4.44	10.44 ± 6.87	0.63	0.004
**Malignant**	9	16.20 ± 12.35	15.88 ± 13.63	0.93	

*P* < .05 of the t-test was considered statistically significant. ICC-values are calculated from the averages of all ROI sizes and planes.

### Differentiation of benign and malignant LNs using SWE data

**Fig 4:**
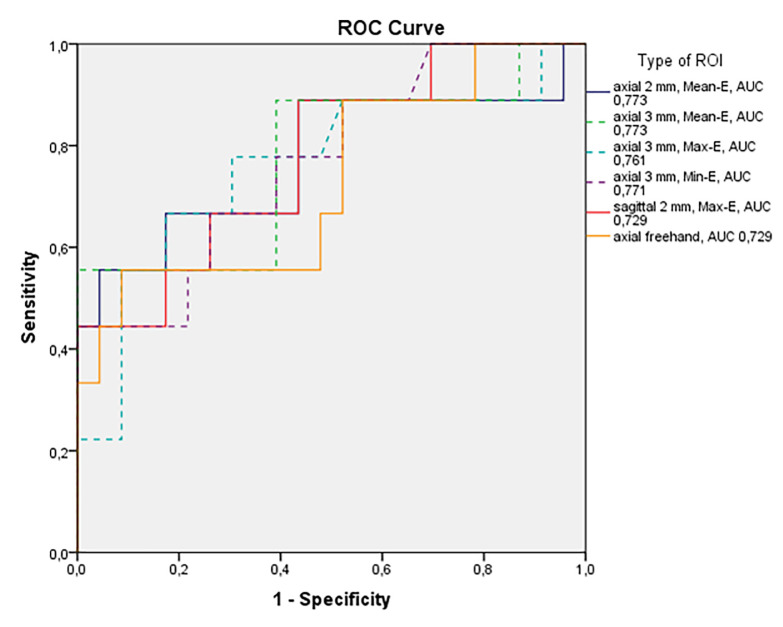
Receiver operating characteristic (ROC) curves of SWE ROIs with the highest AUC values. Axially placed 2 mm and 3 mm ROIs yielded the best results with Mean-E (88.9% sensitivity, 56.5% specificity and 88.9%, 60.9% respectively) and 3 mm Max-E (77.8% sensitivity and 69.4% specificity).

When the SWE data was used to differentiate benign from malignant LNs, 2 mm and 3 mm Mean-E ROIs in axial plane yielded both the highest AUC value of 0.773 in the ROC-curve, 2 mm ROI (*P* = 0.018, 95% CI 0.559–0.987) and 3 mm ROI (*P* = 0.018, 95% CI 0.568–0.978) (Fig 4). Optimal Mean-E cut-off values differentiating benign from malignant inguinal LNs from the ROC-curve analysis was 7.31 kPa when measured from 3 mm axial ROI and 6.6 kPa with 2 mm axial ROI. These results corresponded to 88.9% sensitivity, 60.9% specificity, 47.0% PPV, 93.3% NPV and 68.7% accuracy for the detection of a malignant LN with 3 mm axial ROI and 88.9%, 56.5%, 44.4%, 92.8%, 65.6% with 2 mm ROI, respectively. No statistical significance was found in the diagnostic performance between 2 mm and 3 mm ROI (*P* = 1,000).

**Fig 5:**
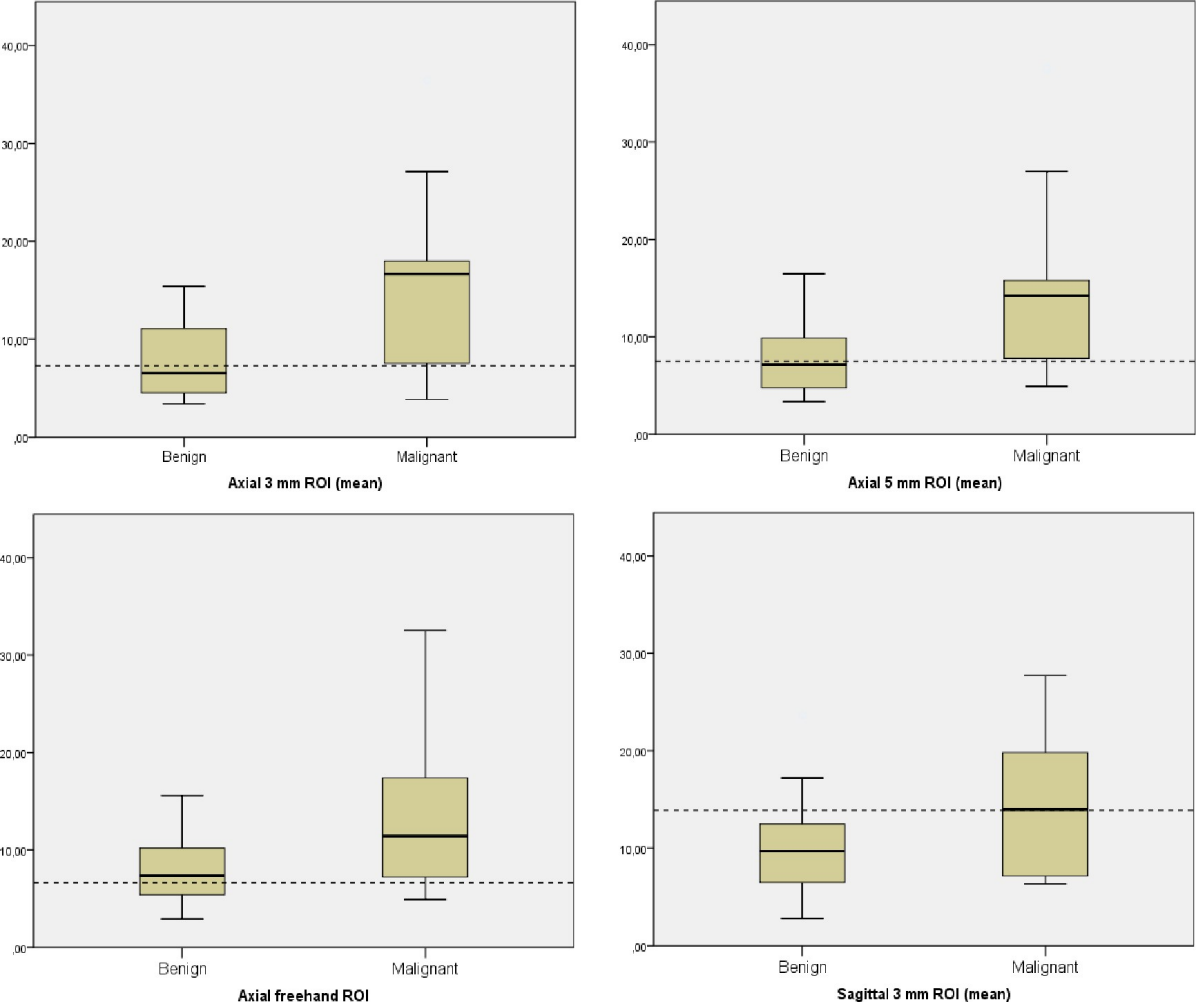
Boxplots of several different types of ROIs. Boxplots of axial 3 mm, 5 mm and freehand ROI and sagittal 3 mm ROIs in benign vs. malignant lymph nodes with the elasticity cut off values (kPa) from the ROC-curve (black dotted line). Axial ROIs demonstrated better diagnostic performance than sagittal ROIs.

Mean-E with 3 mm ROI proved to be higher (15.81 ± 10.61 kPa; range, 3.86–36.45 kPa) in malignant LNs compared to benign LNs (7.68 ± 3.82 kPa; range, 3.41–15.40 kPa). Between the different ROIs with highest AUC-values, there was no significant difference in the diagnostic performance of Mean-E and Max-E (*P* = 0.250). When the diagnostic performance of 3 mm axial and sagittal ROIs were compared, the sagittal ROIs resulted in larger number of false negatives compared to the axial ROIs *(P = 0*.*002)*. Box plots of the Mean-E values in the benign vs. malignant LNs are shown in Fig 5.

## Discussion

The current study demonstrates that SWE measurements in inguinal lymph nodes are reproducible between different observers. This is in congruence with the results from earlier publications where reproducibility and ICC have been found good to excellent in phantom studies and in studies related to SWE in liver and cervical LNs [28–30].

Previously SWE has been mainly used to evaluate axillary and cervical LN regions [2, 3, 7, 15, 31–34]. The first report where SWE was used solely to investigate inguinal LNs was published by Kawahara et al. in patients with metastatic skin cancer [26]. When evaluating suspicious LNs with traditional US, multiple indicators should be assessed. As an adjunct to morphology and vascularity, SWE now provides a new possibility to differentiate malignant from benign LNs. The cut-off value of 7.31 kPa with the 3 mm cortical axially placed ROI resulted in 88.9% sensitivity and 60.9% specificity for differentiating malignancy. The Mean-E values proved to be significantly lower in the benign LNs compared to the malignant LNs. Similar findings of elasticity indices being lower in benign LNs have also been reported axillary and cervical LNs [15, 35]. The result presumably reflects the stiffer nature of the highly cellular metastatic LNs compared to the more loose lymphatic structure of the benign LNs.

Optimal size of ROI for LNs has not been previously investigated. Various ROI sizes and placements have been mostly used in breast masses and cervical LNs. Usually a small 2–3 mm ROI is placed centered in the stiffest part of the lesion [20, 21, 23, 24, 27]. Additionally, placing the ROI to cover the whole suspected lesion has been studied [20, 21, 26]. Since the normal mean cortical thickness in inguinal LNs has been reported to be 1–2 mm [36], we placed ROIs of different sizes to cover majority of the cortex to define the most reproducible ROI size in the inguinal LNs instead of one ROI. In this study, with patients with preoperative US for vulvar cancer, ovarian cancer, lymphoma or patients with otherwise suspected LN enlargement, the mean cortical thickness was 4 mm and the optimal ROI size found to be 3 mm in axial plane.

Smallest ROIs showed more variation in the SWE results than a larger ROIs reflecting the heterogeneity of the tissue. In suspicious LNs, a larger cortical thickness can often be detected in clinical work, allowing use of larger cortical ROI size for SWE. This in turn may average the results, especially with benign LNs consisting of stiffer cortex but also hilar fat leading to lower elasticity values. Due to averaging, our results in inguinal LNs’ Mean-E values differ from breast tissue studies where the mean elasticity remained unchanged between different ROI sizes in benign breast tissue masses [21].

Interrater agreement was good to excellent for Mean-E and Max-E measurements with different ROI sizes. However, if only one ROI size were to be selected for clinical practice, the 3 mm ROI in axial plane proved to be most reliable according to our results. This finding is in line with previous breast tissue findings [20, 21, 24]. ROIs placed on the sagittal plane constantly yielded lower ICC values, reason behind the result remains unclear and further studies are needed to verify this result. The interrater agreement was also better with larger ROIs compared to smaller ones; large 5 mm ROIs resulted in even better ICC-values than 3 mm ROI. The finding can be mainly explained by overlapping and averaging large ROIs in smaller LNs. However, the large 5 mm ROIs yielded a lower sensitivity and specificity since medullar fat might also be included in the larger ROI.

Vicinity of the femoral artery did not affect the results of the elasticity measurements in our cohort. Similar findings have been published regarding the carotid artery and the depth of the LN in neck area [30]. However, in this study superficial LNs resulted in higher reproducibility than the deep LNs. In our cohort the median depth of the superficial inguinal LNs was 15 mm, but with obese patients the measurements may have limited reproducibility. Similar depth dependence has been shown in phantom studies, where it has been shown that the reliability of SWE is reduced especially in hard lesions with depths greater than 2 cm [16, 17]. Although muscle tension and stress from stretching has been found to affect the elastography measurements, they had no effect on the two perpendicular planes in the groin area. Unlike in the neck region where muscle tension may be high, the inguinal region will stay in a relaxed state when the patient is studied in supine position.

In literature the studies of SWE in head & neck and axillary LNs report a sensitivity of 81.0–95.0% and specificity of 80–95% to identify malignant LNs [7, 10, 14, 15, 31]. The results of the present study with suspicious inguinal LNs yielded similar sensitivity but a lower specificity. However, the relatively low number (9 cases) of malignant LNs and heterogenous patient material in this study may influence the result. Only one small study has been conducted solely on inguinal LNs. However, due to its small study population no sensitivity or specificity numbers were given [26]. Other previous studies have had superficial LNs from different areas [33]. On the other hand, this is also the case in clinical setting with suspicious inguinal LNs.

In this study the Mean-E cut-off value for malignant LNs was 7.31 kPa when using the 3 mm ROI. The earlier reports for SWE in inguinal LN diagnosis are scarce, but in head & neck and axillary LN studies the cut-off values have been reported to be higher, between 20 to 56 kPa [31, 32, 35, 37, 38]. None of these LN SWE studies were conducted with similar US equipment as the present study and it is evident that the elasticity values may differ between the US manufacturers. Furthermore, the higher elasticity values in the previous studies are based on a single ROI placed in the stiffest part of the LN which leads to higher elasticity values. This may be due to the finding that placing a ROI over the stiffest lesion seems to increase the specificity of the measurements [7, 10, 14, 31].

Our study has some limitations. As a single center study, the cohort was small and had relatively low number of malignant LNs. Additionally patients had various background diseases which might affect the SWE results. Multicenter studies with a larger cohort and malignant LNs are needed to confirm the results of the present preliminary study. Our results on the usefulness of ROI selection are based only on the Logiq E9 ™ US-device. Since the SWE results may differ between US devices, further studies with different manufacturers’ US devices are needed to verify the results. Furthermore, although the SWE technique might be reproducible in patients with low and normal weight, the inguinal region with obese people is challenging due to skinfolds and may lead to excess pressure of the US probe against skin thus potentially influencing the result of the elasticity measurement. Due to software issues some elasticity indices, including maximum elasticity in the ROI, were not automatically included in all of the measurements. Thus, maximum elasticity index could not be used. In the future, a study with the maximum elasticity index may improve the results and help to better set the optimal cut off values for better differentiation between benign and malignant inguinal LNs.

## Conclusion

This prospective study shows that 2D-SWE provides a reproducible tool for multiparametric, noninvasive US assessment in inguinal LNs. The most reproducible way to measure 2D-shear wave elastography in inguinal LNs is a 3 mm circular ROI centered on the cortex of the LN in axial plane. Further studies, with larger populations with benign and malignant LNs, are needed to evaluate the cut-off values for SWE lymph node elasticity measurements.

## Abbreviations

CNB: core needle biopsy
E: elasticity
ICC: intraclass correlation coefficient
LN: lymph node
ROI: region of interest
SWE: shear wave elastography
US: ultrasound
